# Items

**Published:** 1904-09

**Authors:** 


### ITEMS.

CIVIL SERVICE EXAMINATIONS FOR THE STATE AND COUNTY SERVICE.—The state civil service commission announces general examinations to be held September 10, 1904, including the following positions: apothecary in state hospitals and institutions; architectural draughtsman; assistant steam engineer, Erie county service; bridge draughtsman; clerk; junior clerk; fireman; librarian, court of appeals library, Syracuse; physicians in state hospitals and institutions of both regular and homeopathic schools; trained nurse in state hospitals and institutions; women officer in houses of refuge and reformatories for women.

Applications for these examinations must be made on or before September 3. Full particulars of the examinations and application blanks may be obtained by addressing the Chief Examiner of the Commission at Albany.

THE Wabash railroad offers exceptional facilities for visitors to the World's Fair. The rate from Buffalo, for a fifteen-day limited return ticket is $19.75. The Wabash lands the traveler at the fair grounds or at the Union station at Saint Louis, as may be preferred, and furnishes en route first-class Pullman car service, chair cars and a la carte restaurant service on splendid dining cars. Physicians of Buffalo contemplating a visit to the fair, should interview the Wabash representatives before purchasing tickets.

